# Dr. Elbert W. La Wall

**Published:** 1907-04

**Authors:** 


Dr. Elbert W. La Wall, of Scio, N. Y., died at his home in that village February 22, 1907, of heart disease, aged 33 years. He graduated in medicine at the University of Buffalo in 1897.

